# Saponin-permeabilization is not a viable alternative to isolated mitochondria for assessing oxidative metabolism in hibernation

**DOI:** 10.1242/bio.011544

**Published:** 2015-05-15

**Authors:** Katherine E. Mathers, James F. Staples

**Affiliations:** Department of Biology, University of Western Ontario, London, Ontario N6A 5B7, Canada

**Keywords:** Metabolism, Hibernation, Mitochondrial respiration, Metabolic suppression, Permeabilized tissue

## Abstract

Saponin permeabilization of tissue slices is increasingly popular for characterizing mitochondrial function largely because it is fast, easy, requires little tissue and leaves much of the cell intact. This technique is well described for mammalian muscle and brain, but not for liver. We sought to evaluate how saponin permeabilization reflects aspects of liver energy metabolism typically assessed in isolated mitochondria. We studied the ground squirrel (*Ictidomys tridecemlineatus* Mitchell), a hibernating mammal that shows profound and acute whole-animal metabolic suppression in the transition from winter euthermia to torpor. This reversible metabolic suppression is also reflected in the metabolism of isolated liver mitochondria. In this study we compared euthermic and torpid animals using saponin permeabilized tissue and mitochondria isolated from the same livers. As previously demonstrated, isolated mitochondria have state 3 respiration rates, fueled by succinate, that are suppressed by 60-70% during torpor. This result holds whether respiration is standardized to mitochondrial protein, cytochrome a content or citrate synthase activity. In contrast, saponin-permeabilized liver tissue, show no such suppression in torpor. Neither citrate synthase activity nor VDAC content differ between torpor and euthermia, indicating that mitochondrial content remains constant in both permeabilized tissue and isolated mitochondria. In contrast succinate dehydrogenase activity is suppressed during torpor in isolated mitochondria, but not in permeabilized tissue. Mechanisms underlying metabolic suppression in torpor may have been reversed by the permeabilization process. As a result we cannot recommend saponin permeabilization for assessing liver mitochondrial function under conditions where acute changes in metabolism are known to occur.

## INTRODUCTION

Mitochondria transform chemical energy obtained from the environment into ATP that can be utilized by animal cells for development, growth, survival and reproduction. Many animals are periodically faced with environmental conditions that constrain the ability of mitochondria to fulfill this role. For example seasonal changes in temperature, sunlight and water may limit the amount of energy available for animals. Reversible suppression of oxidative phosphorylation is a strategy used by many organisms to conserve energy under such natural environmental stresses. Understanding the mechanisms that underlie this mitochondrial metabolic suppression will offer great insights into how these animals survive in extreme conditions, and has been the focus of many studies (e.g. Galli and Richards, 2012). In addition, changes in mitochondrial metabolism contribute to many pathological conditions including myopathies (Kunz et al., 1993), neuropathies (Yao et al., 2009), and liver cirrhosis (Krähenbühl et al., 2000). For these reasons accurate analysis of mitochondrial function is crucial for both basic and applied and research.

Oxidative metabolism has been assessed using mitochondria isolated from tissues by homogenization and differential centrifugation, at least as early as 1955 (Chance and Williams, 1955). For fibrous animal tissue (e.g. skeletal muscle), homogenization is often combined with mild proteolytic digestion (e.g. Chance and Williams, 1955). Some analyses may require further purification from these “crude” mitochondrial fractions, for example, by density gradient centrifugation (Petit et al., 1990). These techniques have been helpful in characterizing many aspects of mitochondrial metabolism but, as pointed out by Kuznetsov et al. (2008) they have several limitations. Mitochondrial isolation involves mechanical homogenization, which may alter mitochondrial morphology and interactions with other cellular components (e.g. cytoskeleton, endoplasmic reticulum), and perhaps impact function. Some methods of mitochondrial isolation preferentially retain certain mitochondrial subpopulations (Krieger et al., 1980), potentially biasing results, especially when extrapolating conclusions to higher levels of organization. In addition, mitochondrial isolation requires substantial amounts of tissue, a costly refrigerated centrifuge and a considerable amount of time and skill. As a result of these limitations, many researchers have opted recently to analyze mitochondrial function in permeabilized tissue slices.

To achieve permeabilization, tissues typically undergo fairly gentle mechanical disruption, either by slicing or separation using fine forceps, followed by incubation with steroid-containing compounds such as saponin. Because of its high affinity for cholesterol, saponin binds to cholesterol within plasma membranes, causing it to aggregate, thereby creating fairly large pores in the membrane (Kuznetsov et al., 2008). These pores allow diffusion of relatively small molecules (e.g. pyruvate, succinate, ADP) from the incubation medium to the mitochondria within otherwise intact cells. Because mitochondrial membranes contain much less cholesterol than the plasma membrane (reviewed by Yeagle, 1985), brief treatment with saponin should not uncouple mitochondrial oxidative phosphorylation. In fact, an early study found that oxidative phosphorylation in saponin-permeabilized muscle fibers was almost identical to mitochondria isolated from the same tissue (Kunz et al., 1993). In addition, maximal respiration rates showed very good correspondence between the two techniques.

In recent years the use of saponin permeabilization has expanded greatly and, in conjunction with high-resolution respirometry, has been used to analyze mitochondrial metabolism in skeletal (Casas et al., 2008) and cardiac muscle (Galli and Richards, 2012), gastric mucosa (Gruno et al., 2008), and brain tissue (Benani et al., 2009; Clerc and Polster, 2012). It is particularly useful for small organisms such as *Drosophila* (Pichaud et al., 2010), and small biopsies from larger animals, including humans (Brands et al., 2011). However, while “mechanical permeabilization” has been used to assess mitochondrial function in liver biopsies (Kuznetsov et al., 2002), to our knowledge, no study has used saponin or similar compounds to permeabilize liver tissue for mitochondrial studies. Therefore, the first goal of our study was to assess the utility of saponin permeabilization for analysis of liver mitochondrial metabolism. Similar to other tissues, mammalian liver mitochondria contain little cholesterol relative to phospholipid, so we predicted that brief incubation with saponin would disrupt plasma membranes without uncoupling mitochondrial oxidative phosphorylation. We employed a similar strategy to Kunz et al. (1993) and compared small liver slices, permeabilized using saponin, with mitochondria isolated from the remaining liver tissue of the same animal.

A further goal of this study was to evaluate whether saponin permeabilization of liver is an appropriate technique for assessing the mitochondrial metabolism during whole-animal metabolic states that are known to change the function of isolated mitochondria. Saponin permeabilization of rat brain has been used to assess mitochondrial function among some physiological conditions, such as fasting, that are known to alter whole-animal metabolism over fairly long time periods (i.e. days to weeks; Kuznetsov et al., 2008). However, this technique may not be appropriate for other tissues or conditions where metabolism changes acutely. For example, in cardiac muscle, the activities of some mitochondrial enzymes differ depending on whether they are assayed in isolated mitochondria or tissue biopsies (Phillips et al., 2012). We decided, therefore, to use our well-characterized mammalian hibernation model to evaluate the utility of the saponin permeabilization technique for evaluating changes in liver mitochondrial metabolism.

Throughout the late autumn and winter, ground squirrels undergo bouts of torpor, characterized by low and constant body temperature (T_b_; approximately 5°C) and metabolic rate (<10% of euthermic rates) for several days. These bouts are spontaneously interrupted every 7–12 days by arousals, during which T_b_ and metabolic rate rapidly increase over several hours. Once T_b_ reaches approximately 37°C during an arousal, metabolic rate and T_b_ stabilize for approximately 8 h; a period referred to as interbout euthermia (IBE). IBE is followed by entrance into another bout of torpor when metabolic rate and T_b_ decline again over a few hours. In thirteen-lined ground squirrels (*Ictidomys tridecemlineatus* Mitchell), whole-animal metabolic rate decreases by over 90% in the short time it takes to enter a bout of torpor. This drop in whole-animal metabolism corresponds with a ∼70% suppression of succinate-fueled state 3 respiration of isolated liver mitochondria, even when assayed at a constant *in vitro* temperature (37°C) (Brown et al., 2012).This natural model of metabolic plasticity allowed us to investigate the effectiveness of the saponin permeabilization technique for evaluating changes in liver mitochondrial function. We predicted that the large decreases in respiration rates seen in isolated mitochondria during torpor would be paralleled by respiration rates measured in saponin-permeabilized liver tissue.

## RESULTS

Permeabilized tissue and mitochondria isolated from IBE animals showed strong respiratory control, increasing respiration rate up to 6-fold upon addition of ADP when succinate was used as an oxidative substrate (supplementary material Fig. S1). The mass of tissue slices treated with saponin ranged from 4.0–12.1 mg, but mass-specific state 3 respiration rate was independent of tissue slice mass (supplementary material Fig. S2), indicating that the saponin treatment yielded similar effects across this mass range.

**Fig. 1.**
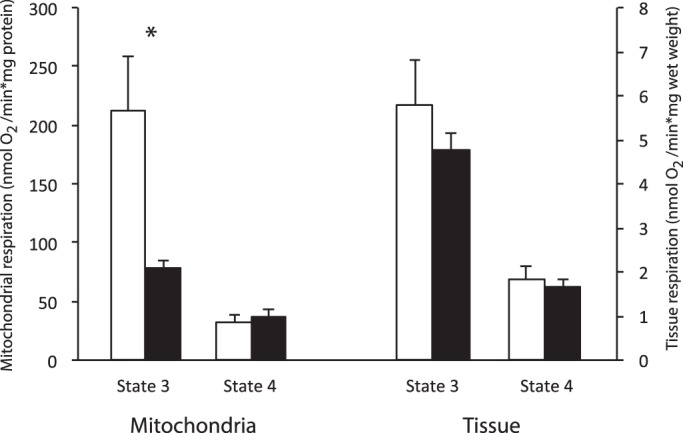
**Succinate oxidation in isolated liver mitochondria and permeabilized liver tissue.** Liver was taken from squirrels during IBE (white bars) and torpor (black bars). Both state 3 and state 4 rates are shown. Respiration rates of isolated mitochondria were standardized to protein concentration, and for permeabilized tissue respiration were standardized to wet weight. Values represent means±s.e.m. Sample sizes for tissue slices are 5 (IBE) and 4 (torpor). Sample sizes for mitochondria are 5 (IBE) and 5 (torpor). *P<0.05.

Succinate-fuelled state 3 respiration rates of isolated mitochondria in torpor was suppressed by 60% when compared with IBE. This metabolic suppression was not reflected in saponin-permeabilized tissue taken from the livers of the same animals (Fig. 1). State 3 respiration did not differ between IBE and torpor in either permeabilized liver or isolated mitochondria when either glutamate or pyruvate was supplied as substrate (Table 1). There were no differences between state 4 rates during torpor and IBE for any of the substrates in either isolated mitochondria or permeabilized tissue (Table 1 and Fig. 1).

**Table 1.**
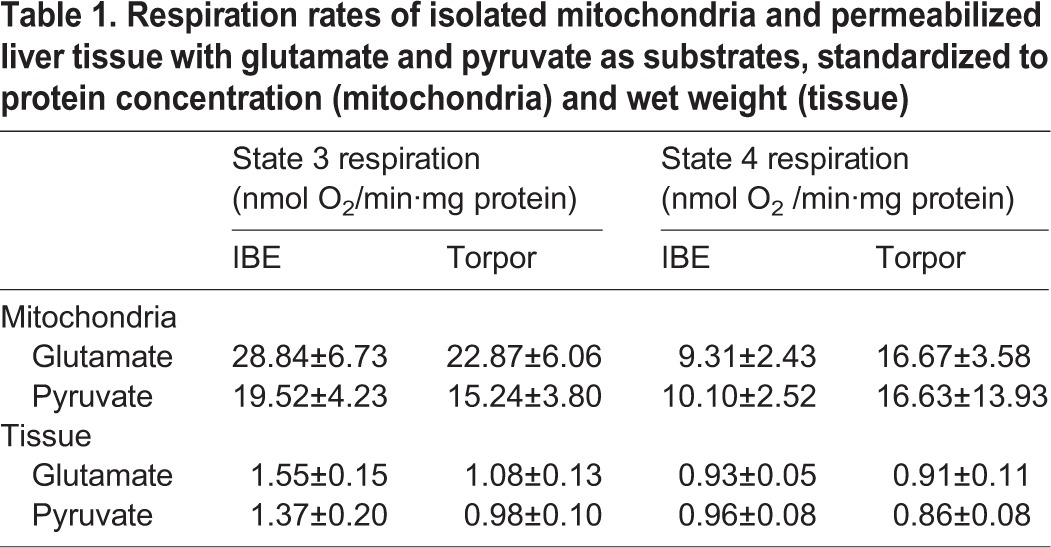
**Respiration rates of isolated mitochondria and permeabilized liver tissue with glutamate and pyruvate as substrates, standardized to protein concentration (mitochondria) and wet weight (tissue)**

**Fig. 2.**
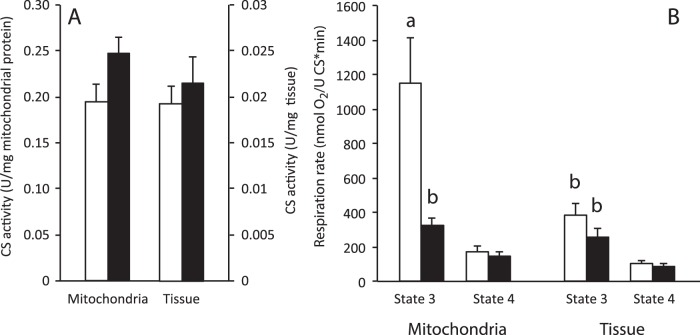
**Citrate synthase activity in isolated liver mitochondria and permeabilized liver tissue (A) and state 3 respiration with succinate standardized to citrate synthase activity (B).** Mitochondria were isolated and tissue was permeabilized from livers of IBE (white bars) and torpid (black bars) ground squirrels. Sample sizes for mitochondria are 5 (IBE) and 5 (torpor). Sample sizes for permeabilized tissue are 5 (IBE) and 5 (torpor). Bars represent means±s.e.m. Within state 3 respiration rates, bars that do not share the same letter label are significantly different from each other. Differences were considered significant at p<0.05.

We measured citrate synthase activity to assess the possibility that the respiratory suppression seen in isolated mitochondria from torpid animals was due to changes in mitochondrial abundance, or decreases in protein content not related to oxidative phosphorylation. Citrate synthase activity did not differ between torpor and IBE in either isolated mitochondria or permeabilized tissue (Fig. 2A). When standardized to citrate synthase activity, respiration rates with succinate in isolated mitochondria showed the same pattern as the rates standardized to protein concentration; during torpor, rates were suppressed by 70% (Fig. 2B). In permeabilized tissue, however, state 3 respiration rates did not differ between torpor and IBE. Assays of citrate synthase in both permeabilized tissues and isolated mitochondria also provided a common denominator for expressing respiration rates, allowing us to directly compare the two techniques. When standardized to citrate synthase activity, state 3 respiration rates in permeabilized slices from IBE animals were significantly lower than isolated mitochondria (Fig. 2B). There were no differences between state 3 rates in torpor and IBE when either glutamate or pyruvate was used as substrate (Table 2). There were no differences between state 4 rates in torpor and IBE for either isolated mitochondria or permeabilized tissue for any of the substrates (Table 1).

**Table 2.**
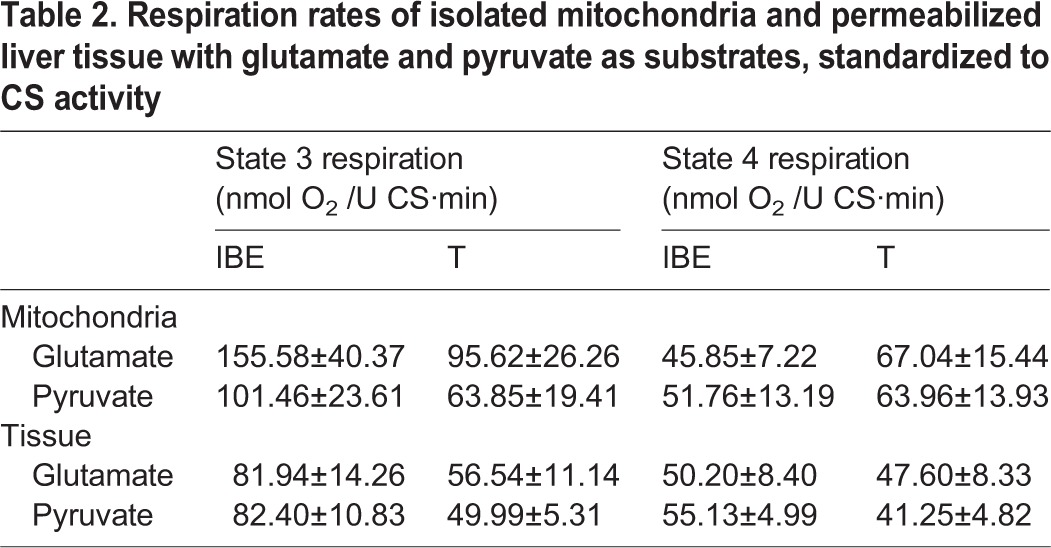
**Respiration rates of isolated mitochondria and permeabilized liver tissue with glutamate and pyruvate as substrates, standardized to CS activity**

**Fig. 3.**
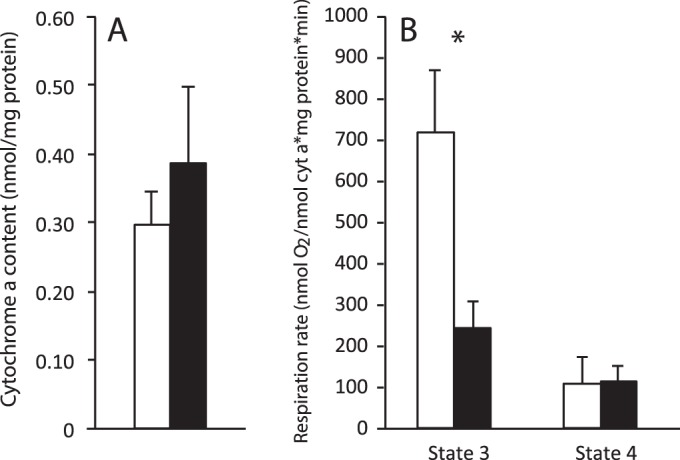
**Cytochrome a concentration in isolated mitochondria.** Cytochrome a content was measured in isolated mitochondria in IBE and torpid ground squirrels (A). State 3 respiration of isolated mitochondria with succinate as substrate, standardized to cytochrome a content, is also shown (B). Bars represent means±s.e.m. Sample sizes are 5 (IBE) and 5 (torpid). *P<0.05.

We attempted to further assess potential changes in protein or mitochondrial content between IBE and torpor by measuring cytochrome a content. In isolated mitochondria cytochrome a content did not differ between torpor and IBE (Fig. 3A). When standardized to cytochrome a content, state 3 respiration with succinate in torpor again showed a 70% suppression, compared with IBE (Fig. 3B). In tissue slices, however, the cytochrome a content could not be determined (see Discussion, below).

**Fig. 4.**
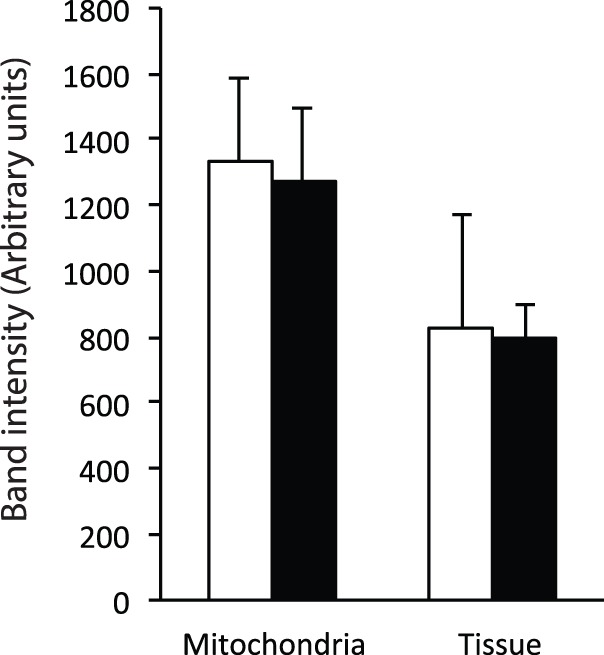
**VDAC content in isolated liver mitochondria and permeabilized liver slices.** Band intensities of VDAC immunoblots from mitochondria and permeabilized slices from liver tissue of IBE (white bars) and torpid (black bars) ground squirrels. Sample size for mitochondria is 3 (IBE) and 3 (torpor). Sample size for permeabilized tissue is 3 (IBE) and 3 (torpor). Bars represent means±s.e.m.

We also measured voltage-dependent anion channel (VDAC) content as an indicator of mitochondrial content. There was no significant difference in VDAC content between torpor and IBE in either isolated mitochondria and permeabilized liver (Fig. 4), indicating that total mitochondrial content does not change between torpor and IBE.

**Fig. 5.**
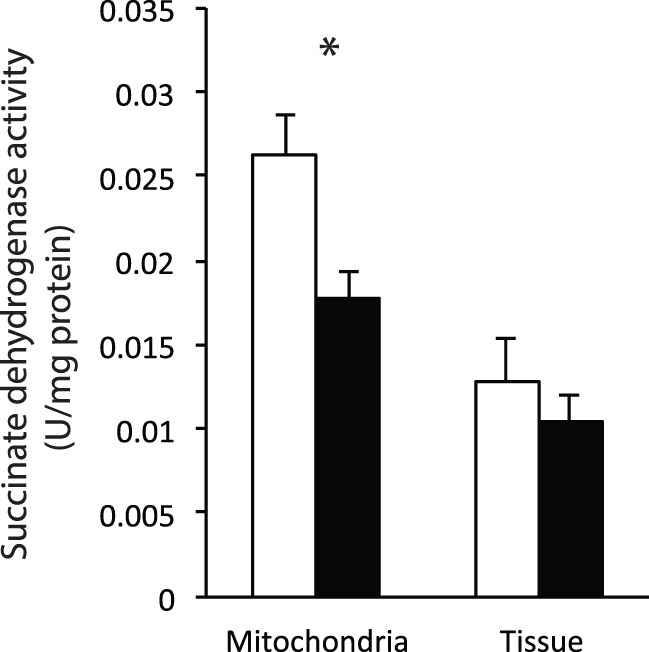
**Succinate dehydrogenase activity in isolated liver mitochondria and permeabilized liver slices.** Rates are shown for ground squirrels during IBE (white bars) and torpor (black bars). Bars represent means±s.e.m. Sample size for mitochondria is 3 (IBE) and 3 (torpor). Sample size for permeabilized tissue is 4 (IBE) and 3 (torpor). *P<0.05.

Succinate dehydrogenase (SDH) activity was reduced by 33% during torpor when measured in isolated mitochondria, but there was no difference between torpor and IBE in permeabilized liver (Fig. 5).

## DISCUSSION

The first goal of this study was to assess how suitable saponin permeabilization is for studying mitochondrial metabolism in mammalian liver. In comparison with an earlier study using mechanically permeabilized pig liver (Kuznetsov et al., 2002), our ground squirrel liver yielded state 3 respiration rates fueled by succinate that were slightly higher and respiratory control ratios (state 3/state 4) that were slightly lower. These differences are to be expected, however, given the allometric relationship of body mass with both hepatocyte respiration (Porter and Brand, 1995) and mitochondrial proton leak (Porter et al., 1996), and the difference in body mass between pigs and ground squirrels. When supplied with oxidative substrates – especially succinate – permeabilized ground squirrel liver tissue displayed robust respiratory control, with rapid and substantial increases in oxygen consumption upon addition of ADP that were virtually eliminated upon addition of oligomycin (supplementary material Fig. S1).

The second goal of this study was to assess the utility of saponin permeabilization for evaluating mitochondrial metabolism in metabolic states that are known to change the metabolism of isolated mitochondria. Tissue permeabilization has previously been employed in rat brain tissue to assess mitochondrial function in relation to physiological conditions that affect whole-animal metabolism, such as fasting (Benani et al., 2009). In the current study, state 3 respiration rates, fueled by succinate, were 60–70% lower in mitochondria isolated from torpid animals compared with IBE. It is possible that our purification procedure preferentially retained mitochondria from torpid animals that have low respiration rates, considering homogenization of rat liver yields different populations of mitochondria with varying respiration rates (Lopez-Mediavilla et al., 1989). We feel, however, that is an unlikely explanation for our data, as several previous studies (e.g. Barger et al., 2003; Muleme et al., 2006) found comparable suppression of succinate-fueled state 3 respiration in torpor using crude mitochondrial fractions that, presumably, contain a mixture of mitochondrial populations. We examined the ratio of succinate dehydrogenase (SDH; a membrane enzyme) to citrate synthase (CS; a matrix enzyme) activity to assess the possibility that the isolation process compromises mitochondrial structural integrity. When comparing isolated mitochondria and permeabilized liver tissue, we found a significantly higher SDH:CS ratio in mitochondria (0.15 in mitochondria and 0.08 in permeabilized tissue; *P*=0.02). This may indicate that the structural integrity of mitochondria is affected by the isolation process, resulting in a loss of matrix proteins. We believe, however, that this observation is unlikely to explain our observed differential response of mitochondria and permeabilized tissue to torpor for at least two reasons. First, succinate oxidation does not depend on CS or any other matrix enzymes, so any loss during mitochondrial isolate should not affect succinate oxidation. Second, in isolated mitochondria, there is significant suppression whether the rates are expressed relative to mitochondrial protein, cytochrome a, or CS; if CS is differentially lost between torpor and IBE, it is unlikely that total protein and cytochrome a are as well.

It is also possible that differences in media between the two preparations may have contributed to the different patterns of respiratory suppression; notably, the presence of ATP in the medium used for permeabilized tissue may have inhibited mitochondrial function. We do not believe this is likely, however, since the respiratory rates reported in this study are higher than those reported in a study of mechanically permeabilized pig liver that used a media that did not contain ATP (Kuznetsov et al., 2002). Nonetheless we feel that it may be beneficial to conduct a similar study with and without the presence of ATP in the permeabilization isolation medium.

Earlier hibernation studies (Barger et al., 2003; Muleme et al., 2006) expressed mitochondrial respiration rates relative to protein content, leaving the possibility that differences between IBE and torpor were due to changes in the protein content of mitochondrial preparations. In this study we also expressed the respiration rates of isolated mitochondria relative to two independent mitochondrial markers, cytochrome a content and citrate synthase activity, and found a similar degree of respiratory suppression in torpor. These novel findings confirm solidly the existence of a reversible suppression of mitochondrial metabolism in hibernation. In permeabilized liver slices, however, state 3 respiration did not differ between IBE and torpor (Figs 1 and 2).

Since we expressed respiratory rates for both isolated mitochondria and permeabilized tissue relative to a common denominator, citrate synthase activity, it is unlikely that the lack of suppression seen in permeabilized tissue was due to changes in mitochondrial content between torpor and IBE. Citrate synthase activity is considered a good marker for total mitochondrial content (Larsen et al., 2012), and did not change in permeabilized tissue between torpor and IBE. We also found that cytochrome a content, another good marker of mitochondrial content (Larsen et al., 2012), did not differ in isolated mitochondria between experimental groups, suggesting that the content of electron transport system complexes does not change acutely between topor and IBE. Unfortunately we were unable to quantify cytochrome a content in permeabilized tissue because the spectrum was masked by some tissue contaminant, presumably hemoglobin, although no clotted blood was apparent. To prevent such contamination we recommend perfusing tissue with heparinized buffer immediately after euthanasia. Nonetheless, the fact that neither citrate synthase activity nor VDAC content changed between torpor and IBE, in either isolated mitochondria or permeabilized liver, allow us to conclude with confidence that total mitochondrial content does not change between torpor and IBE.

Expressing respiration rates relative to a common denominator (citrate synthase activity) also allowed us to compare directly respiration rates between isolated mitochondria and permeabilized tissue. State 3 respiration rates fueled by succinate were quite similar in isolated mitochondria from torpid animals and permeabilized tissue from both IBE and torpid animals. In IBE animals, however, these rates were almost 3-fold higher in isolated mitochondria compared with permeabilized tissue from the same animals. It is possible, therefore, that the lack of suppression that we observed in permeabilized tissue actually reflects some constraint on the ability of permeabilized tissue to achieve very high rates of oxygen consumption. Our experimental design did not allow us to determine the nature of any such constraints, and we used concentrations of substrates and O_2_ that yielded maximal respiration rates in permeabilized tissue. Although we believe that other factors likely led to the observed result (see following paragraph) we advise caution when using permeabilized tissue for assessing very high respiration rates.

It is also possible that the differences observed between isolated mitochondria and permeabilized tissue are due to the permeabilization procedure causing reversal of processes responsible for mitochondrial suppression during torpor. We tested this hypothesis by comparing the activity of succinate dehydrogenase (SDH) between IBE and torpor in isolated mitochondria and permeabilized tissue. In accordance with previous studies (Armstrong and Staples, 2010; Chung et al., 2011), SDH activity was significantly suppressed during torpor in isolated mitochondria. In permeabilized tissue, however, there was no significant difference in SDH activity between IBE and torpid animals. This indicates that SDH activity may be an important factor in metabolic suppression during torpor, and that the mechanisms that control its suppression are likely reversed during the permeabilization process. We are currently exploring how differential phosphorylation and acetylation of mitochondrial proteins might underlie the metabolic suppression seen in these hibernators. It is possible that, during the isolation procedure, homogenization and centrifugation separate mitochondria from the enzymes and cytoskeletal structures required to reverse these suppressing mechanisms. However, the permeabilization process does not completely disrupt cellular structure and may allow for reversal once the liver is removed from the whole-animal environment. Comparing the phosphorylation and acetylation states between isolated mitochondria and permeabilized slices in future studies may reveal the importance of these modifications.

An earlier study (Gallagher and Staples, 2014) used saponin permeabilization to assess the potential for mitochondrial metabolic suppression in hibernation using both brain cortex and cardiac muscle. A subsequent study (Brown and Staples, 2014) using isolated cardiac muscle mitochondria did find significant (approx. 30%), suppression of state 3 respiration, at least with succinate as a substrate. Although these two studies used cardiac muscle from different animals, combined the data support our current observation that saponin permeabilization masks mitochondrial metabolic suppression. These results also suggest that it would be worthwhile to reevaluate the potential for metabolic suppression during torpor in ground squirrel brain using isolated mitochondria.

In summary, we found that saponin permeabilized liver tissue displayed respiratory control comparable to isolated mitochondria from euthermic ground squirrels; however, permeabilized tissue from torpid ground squirrels did not display the suppression of state 3 respiration seen in the isolated mitochondria from torpid animals. In addition we found that, while activity of succinate dehydrogenase was suppressed during torpor in isolated mitochondria, there was no significant suppression in permeabilized tissue. We conclude, therefore, that the saponin permeabilization technique is of limited utility for comparing liver mitochondrial function among conditions known to alter acutely whole-animal metabolism unless the effects of these conditions of tissue composition and mitochondrial enzymes are characterized.

## MATERIALS AND METHODS

### Animals

Thirteen-lined ground squirrels (*Ictidomys tridecemlineatus*) were live-trapped in Carman, MB, Canada (49°29′57″N, 98°0′3″W). Animals were housed individually in plastic shoebox-style cages (26.7 cm×48.3 cm×20.3 cm high) with dried corn cob bedding, paper nesting material (Crinkl-l'Nest, The Andersons, Inc.), wooden chewing sticks (to control incisor growth), and a transparent red plastic tube (Bio-Serv, Frenchtown NJ) for enrichment. Rat chow (LabDiet 5P00), dry dog food (Iams), and water were provided *ad libitum*, with sunflower seeds and corn provided three times a week. Each animal was weighed during weekly cage cleaning.

Squirrels were housed at 22°C±3°C and a photoperiod matching that of Carman, MB (adjusted weekly) until October. In early October, animals were moved to environmental chambers where the temperature was reduced 1°C/day until it reached 4°C±2°C. At this time photoperiod was reduced to 2 h of light and 22 h of dark, with lights on at 09:00 h. Food and water were provided *ad libitum* until torpor was observed (typically within one week), at which time food was withdrawn, because this species does not eat throughout hibernation season. Hibernation state was determined by T_b_, which was monitored continuously using implanted radio telemeters (Data Sciences International, St. Paul, MN), as described previously (Muleme et al., 2006).

In this study we compared animals that were either in torpor (a stable T_b_ near 5°C for 3-5 days) or IBE (stable T_b_ near 37°C for 3–4 h following a spontaneous arousal). IBE squirrels were euthanized by anaesthetic overdose (Euthanyl, 270 mg/ml, 0.2 ml/100 g), but torpid squirrels were euthanized by cervical dislocation, as anaesthetic injection would cause arousal. Euthanyl is not known to affect mitochondrial metabolism (Takaki et al., 1997). There was no significant difference in body mass between the IBE and torpid groups.

### Permeabilization of liver tissue

Our permeabilization technique incorporated saponin with a previously described mechanical disruption technique (Kuznetsov et al., 2002). Following euthanasia, a small (∼100 mg) portion of left liver lobe was transferred into ice cold isolation buffer (20 mM taurine, 20 mM imidazole, 0.5 mM DTT, 10 mM Ca^2+^EGTA, 5.77 mM ATP, 6.56 mM MgCl_2_, 57.5 mM K^+^-MES, pH 7.0), while the rest of the liver was reserved for mitochondrial isolation. This buffer was modified from that used for permeabilization of mammalian brain (Benani et al., 2009) and muscle (Kuznetsov et al., 2008) by omitting phosphocreatine, as liver does not express creatine kinase (Meffert et al., 2005). The outer serosal capsule of the liver was removed, and the inner portion of the liver tissue was sliced into smaller portions of approximately 15 mg (∼1 mm across) using a single-sided razor blade.

Liver tissue slices were placed individually into wells of 12-well plates containing 3 ml of ice-cold isolation buffer, covered and gently agitated (30 rpm) on ice using a metabolic shaker. Following 5 min of agitation, 2.5 μl of freshly prepared saponin (Sigma) solution (5 mg/ml isolation buffer) was added to each well. Following the addition of saponin, agitation continued for an additional 20 min. The concentration of saponin and exposure time were empirically optimized in preliminary experiments to yield the highest respiratory control ratio (state 3/state 4), and were similar to those used in the preparation of mammalian brain (Benani et al., 2009), cardiac muscle (Galli and Richards, 2012), and skeletal muscle (Casas et al., 2008). Following incubation with saponin, tissue slices were gently transferred to 3 ml of ice-cold mitochondrial respiration buffer (0.5 mM EGTA, 3 mM MgCl_2_, 60 mM K-lactobionate, 20 mM taurine, 10 mM KH_2_PO_4_, 20 mM HEPES, 110 mM sucrose, 1 g/l fatty acid free BSA, pH 7.1 (Kuznetsov et al., 2008)) and agitated for 5 min. This step was repeated twice more to remove any residual saponin. Tissue slices were then immediately used for assessment of mitochondrial respiration (see “Respirometry” below). Additional permeabilized slices were frozen at −80°C for later measurement of enzyme activities.

### Isolation of mitochondria

The remainder of the liver (approximately 4 g) was used immediately for mitochondrial isolation. The tissue was rinsed in 20 ml of ice-cold homogenization buffer (250 mM sucrose, 10 mM HEPES, 1 mM EGTA, 0.5% fatty acid free BSA, pH 7.4) and minced in 20 ml of the same buffer. It was then homogenized using three passes of a loose-fitting (0.4 mm clearance) Teflon pestle at 100 rpm in a 30 ml glass mortar. The homogenate was filtered through one layer of cheesecloth and centrifuged at 1000 ***g*** for 10 min at 4°C in polycarbonate centrifuge tubes. Floating lipid was removed by aspiration and the supernatant was filtered through four layers of cheesecloth and centrifuged again at 1000 ***g*** for 10 min at 4°C. Following the removal of lipid this supernatant was centrifuged at 8700 ***g*** for 10 min at 4°C. The supernatant was discarded and any lipid adhering to the tube was carefully removed using cotton swabs. The remaining pellet was resuspended in approximately 20 ml of homogenization buffer and centrifuged at 8700 ***g*** for 10 min at 4°C. Again, the supernatant and adhering lipid were removed, and this final pellet, containing a crude mitochondrial fraction, was resuspended in 1 ml ice-cold homogenization buffer.

The crude mitochondrial fraction was purified using Percoll density gradient centrifugation (Petit et al., 1990). Ten ml each of 10, 18, 30 and 70% Percoll solution (in homogenization buffer) were carefully layered in a 55 ml centrifuge tube. The crude mitochondrial fraction, suspended in 1 ml of homogenization buffer, was carefully layered on top of the Percoll and centrifuged at 13,500 ***g*** for 35 min at 4°C. Mitochondria settled in a visible band between the 30 and 70% Percoll layers. This band was resuspended in 40 ml of homogenization buffer and centrifuged twice at 8700 ***g*** for 10 min at 4°C to remove any residual Percoll. The final, purified mitochondrial pellet was resuspended in 1 ml of ice-cold mitochondrial respiration buffer. Mitochondria were used immediately for assessment of mitochondrial respiration, and an aliquot was frozen at −80°C for subsequent assays.

### Respirometry

The wet mass of permeabilized liver slices was determined, following blotting on filter paper, using a microbalance (MX5, Mettler Toledo). Liver tissue slices were then transferred to chambers of Oxygraph-2K high-resolution respirometer (Oroboros Instruments, Austria) containing 2 ml of mitochondrial respiration medium under constant stirring (750 rpm) at 37°C. Oxygen partial pressure, sensed by polarographic electrodes, was converted to concentration using DatLab software (version 4.3.2.7, Oroboros Instruments, Austria) following calibration with air-saturated medium. As in other permeabilized tissue preparations (e.g. Kuznetsov et al., 2008), the medium was rendered slightly hyperoxic (approximately 350 nmol/ml, 1.8-fold air saturation) to ensure adequate oxygen supply over the relatively large diffusion distances of tissue slices, compared with isolated mitochondria. Hyperoxygenation was achieved by injecting gaseous oxygen into the gas space above the medium within the chambers while stirring before the lids were closed, rendering the chambers airtight. Oxygen was replenished in the medium whenever the concentration fell below approximately 200 nmol/ml to ensure that respiration was not limited by oxygen availability (Pesta and Gnaiger, 2012).

Separate slices of tissue from the same liver were used to assess respiration with pyruvate (10 mM), glutamate (30 mM; both with 4 mM malate) and succinate (30 mM, with 0.5 µM rotenone in ethanol) as oxidative substrates. These concentrations are similar to those used with other saponin-permeabilized tissue preparations (e.g. Benani et al., 2009) and yielded maximal rates in preliminary trials. Once stable state 2 respiration was reached following substrate addition, ADP and Mg^2+^ (both 5 mM) were added to stimulate state 3 respiration. This high concentration of ADP is required to maximize state 3 over the relatively large diffusion distances of permeabilized tissue preparations (Kuznetsov et al., 2008). Such high ADP concentrations, along with the presence of ATPases in the permeabilized tissue preparations, preclude tissues from reaching true state 4 respiration; therefore, state 4 was estimated by the addition of oligomycin (160 µg/ml in ethanol). Liver slice respiration rates were expressed relative to wet weight and citrate synthase activity (seen “Enzyme Assays” below).

Respiration of isolated liver mitochondria was determined at 37°C in 2 ml of the mitochondrial respiration medium under constant stirring. The respiration medium was equilibrated with room air (approximately 190 nmol/ml) without hyperoxygenation. Respiration rates were determined with pyruvate (1 mM), glutamate (5 mM, both with 1 mM malate) and succinate (6 mM, with 0.5 μM rotenone). State 3 was stimulated with 0.2 mM ADP. State 4 was approximated by addition of oligomycin. Unless stated otherwise, all added compounds were dissolved in mitochondrial respiration buffer. Examples of respiration measurements in both isolated mitochondria and permeabilized liver tissue are shown in supplementary material Fig. S1.

### Enzyme assays

Frozen isolated mitochondria and saponin-treated tissue slices were homogenized in 10 volumes of buffer (20 mM Tris, 1 mM EDTA, 0.1% Triton X-100, pH 7.2) using a glass homogenizer. Homogenate protein content was determined using a Bio-Rad dye reagent, with bovine serum albumin dissolved in homogenization buffer as standards. Mitochondrial homogenates were diluted to 1 mg protein/ml, and permeabilized tissue homogenates were diluted to 2 mg protein/ml. Enzyme activities were assayed at 37°C using a Spectramax plate spectrophotometer (Molecular Devices, Sunnyvale, CA) in a 96-well plate.

Citrate synthase (CS) was assayed following the addition of 10 μl of mitochondrial (1 mg/μl) or tissue (1 mg/μl) homogenate to 250 μl of assay mixture containing 50 mM Tris, 0.1 mM 5,5-dithiobis(2-nitro-benzoic acid) (DTNB), and 0.15 mM acetyl CoA. Parallel reactions were run with and without 0.5 mM oxaloacetate. Absorbance values (412 nm) were collected for 5 min at 37°C, with CS activity calculated from the difference between the rates with and without oxaloacetate, using an extinction coefficient of 13.6 l/(mol·cm).

Succinate dehydrogenase (SDH) activity was measured following the addition of 10 μl mitochondrial/tissue homogenate to 250 μl of assay mixture containing 25 mM K_2_HPO_4_ (pH 7.5), 20 mM succinate, 50 μM dicholorophenolindophenol (DCPIP), 1 mM KCN, 100 μM ATP, and 2 mg/ml BSA. Parallel reactions were run with and without 50 μM decylubiquinone. Absorbance values (600 nm) were collected for 5 min at 37°C, and SDH activity was calculated from the difference between the rates with and without decylubiquinone, using an extinction coefficient of 19.2 l/(mol·cm).

### Cytochrome a determination

Mitochondria were solubilized in 100 mM Na_3_PO_4_, 2% Triton X-100, pH 7. Baseline, oxidized absorbance was measured between 510 and 630 nm in a spectrophotometer (Lambda 3B; Perkin-Elmer). The samples were then reduced using excess sodium hydrosulfite, and absorbance was again measured between 510 and 630 nm. Difference spectra between the oxidized and reduced mitochondrial preparations were calculated, and cytochrome a content was determined from the peak at 605 nm using an extinction coefficient of 12 l/(mol·cm) (Balaban et al., 1996).

### VDAC immunoblots

Samples of both isolated mitochondria and permeabilized tissue were denatured in loading buffer at 80°C for 5 min and electrophoresed on sodium dodecyl sulfate (SDS) polyacrylamide gels (10% acrylamide). Gels were loaded with equal volumes of protein (10 ng for isolated mitochondria; 20 ng for permeabilized tissue). Gels were run at 180 V for 1 h in a running buffer (25 mM Tris, 190 mM glycine, 0.01% SDS), then transferred to polyvinylidene fluoride membranes. Transfers were conducted at 4°C at 100 V for 2 h. After transfer, the membrane was blocked with 5% BSA in TBST (30 mM Tris, 137 mM NaCl, 0.1% Tween-20, pH 7.6) under steady agitation for 2 h and probed with primary antibody for VDAC (Abcam, ab34726) overnight at 4°C. Donkey polyclonal Secondary Antibody to Mouse IgG (Abcam, ab6820) was incubated for 1 h at room temperature under steady agitation. The membrane was washed four times for 15 min in Tris-buffered saline and Tween-20 (TBST). VDAC levels were quantified via a chemiluminescent substrate (Millipore) using a VersaDoc MP5000 imaging system (BioRad). Bands were analyzed using the band analysis tool in ImageLab 3.0 (BioRad).

### Statistical analysis

All statistical analyses were conducted using R. For both isolated mitochondria and permeabilized tissue, one-tailed, unpaired *t*-tests were used to compare protein- and wet weight-standardized respiration rates between torpor and IBE. Two-way ANOVAs followed by Tukey-Kramer HSD tests were used to compare state 3 rates for CS-standardized respiration rates. A two-tailed, unpaired *t*-test was used to compare isolated mitochondrial cytochrome a content between torpor and IBE. For VDAC content, two-tailed, unpaired *t*-tests were used to compare between IBE and torpor in isolated mitochondria and permeabilized tissue. One-tailed, unpaired *t*-tests were used to compare SDH activity between torpor and IBE in both isolated mitochondria and permeabilized tissue. Differences were considered significant for *P*-values <0.05.

## Supplementary Material

Supplementary Material
