# Supplementary material for: Saponin-permeabilization is not a viable alternative to isolated mitochondria for assessing oxidative metabolism in hibernation
Source: Biol Open. 2015 May 15;4(7):858–64. doi: 10.1242/bio.011544 (PMC4571088; doi:10.1242/bio.011544)
Supplement: Supplementary Material [file supp_4_7_858__index.html]

Saponin-permeabilization is not a viable alternative to isolated mitochondria for assessing oxidative metabolism in hibernation — Saponin-permeabilization is not a viable alternative to isolated mitochondria for assessing oxidative metabolism in hibernation — Supplementary Material 

# Saponin-permeabilization is not a viable alternative to isolated mitochondria for assessing oxidative metabolism in hibernation

## BIO011544 Supplementary Material

- Supplementary Material
