# Supplementary material for: Saponin-permeabilization is not a viable alternative to isolated mitochondria for assessing oxidative metabolism in hibernation
Source: Biol Open. 2015 May 15;4(7):858–64. doi: 10.1242/bio.011544 (PMC4571088; doi:10.1242/bio.011544)
Supplement: Supplementary Material [file supp_bio.011544_BIO011544supp.pdf]

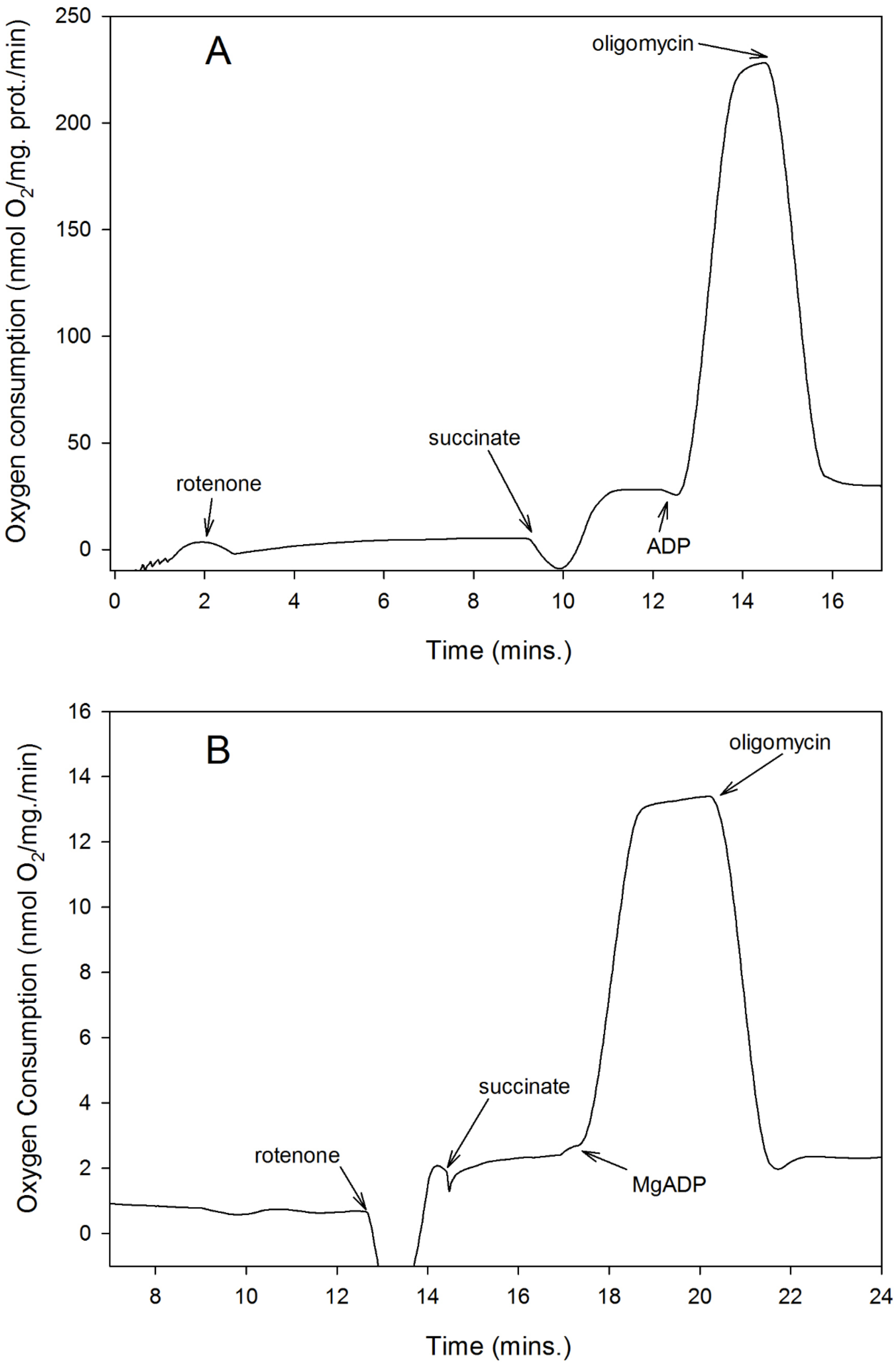

Fig. S1. Examples of succinate oxidation in isolated liver mitochondria (A) and permeabilized liver tissue (B). After stable basal rates were established, rotenone (0.5 µM) and succinate (30 µM) were injected, causing brief injection artifacts. After steady state 2 respiration rates were established, ADP and Mg^{2+} (5 µM) were added to stimulate state 3 respiration. Oligomycin (160 µg/ml) was then added to estimate state 4 respiration.

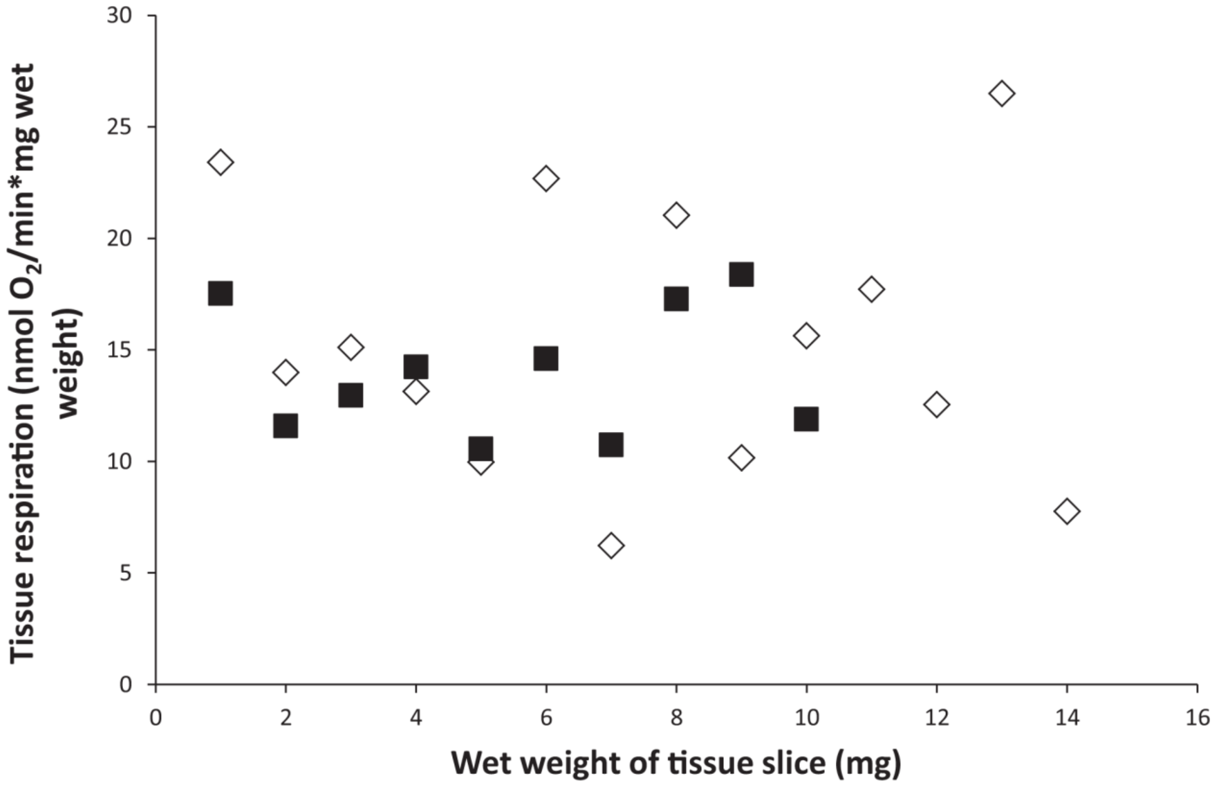

Fig. S2. Succinate oxidation of permeabilized liver slices is independent of slice wet weight. White diamonds represent samples from IBE squirrels (*n*=14) and black squares represent samples from torpid squirrels (*n*=10).
